# Are primary care consultations in Trinidad patient-centered? A cross-sectional study of patients with non-communicable diseases

**DOI:** 10.1186/s12875-023-02149-8

**Published:** 2023-10-21

**Authors:** Reisa R Rahaman, M Shastri Motilal, Raveed Khan, Rohan G Maharaj

**Affiliations:** Faculty of Medical Sciences, Department of Paraclinical Sciences, The University of the West Indies, St. Augustine Campus, St. Augustine, Trinidad

**Keywords:** Patient-centered care, Consultation, Non-communicable disease, Quality, Trinidad

## Abstract

**Background:**

The aim of this study was to measure the patient’s perception of patient centeredness in their consultations for non-communicable diseases (NCDs). We also measured consultation length and patient enablement.

**Method:**

A cross-sectional study was conducted over 2 months at four primary care clinics at the St. Joseph cluster of the North Central Regional Health Authority (NCRHA) in Trinidad and Tobago. Interviewers timed the consultation and completed post-consultation questionnaires using the Patient Perception of Patient-Centeredness (PPPC) questionnaire and the Patient Enablement Index (PEI). The PPPC is a 14-item (each scored 1–4) Likert-scaled instrument. The total score is averaged and a PPPC score of 4 is the maximum. The PEI measures the ability of the patient to cope with life and their disease. The PEI consists of 6 questions scored 0–2, with a maximum score of 12.

**Results:**

There were 180 respondents (response rate = 82.5%). Participants were female (75.6%), aged over 65 years (50.6%), married (51.1%), Indo-Trinidadian (52.2%), and Christian (60.6%). Half achieved a primary school education, and 37.2% secondary. The consultation length ranged between 1.32 and 31.22 min. The average, median and mode of the consultation length were 8.5, 7.74 and 10 min, respectively. The average, median and mode of the measures of patient-centeredness were PPPC (3.67, 3.86 and 4) and PEI score (5.93, 6 and 6). The PPPC average was lower in patients with a stroke (*p* = 0.022), and higher among those with more than 2 consultation interruptions (*p* = 0.015) and those who knew the doctor very well (*p* = 0.015). The PEI score was lower in patients with heart disease (*p* = 0.022). The consultation length was longer in those with tertiary education (*p* = 0.044) and those with two consultation interruptions (*p* = 0.032). PPPC Average and PEI Score correlated well (ρ = 0.408, *p* < 0.001). The consultation length correlated with the PPPC Average (ρ *=* 0.168,*p* = 0.025).

**Conclusion:**

Primary Care consultations in this cluster of health centres in NCRHA in Trinidad were often patient centered. The consultation length, patient-centeredness, measured with the PPPC instrument, and patient enablement scores, measured with the PEI instrument, in consultations for NCDs in Trinidad compare favourably with international reports.

**Supplementary Information:**

The online version contains supplementary material available at 10.1186/s12875-023-02149-8.

Patient-centered care (PCC) is defined as “the use of adequate consulting skills in identifying patients’ priorities and concerns and the appropriate involvement of patients in making decisions about their care” [1]. In this approach the patient’s beliefs and characteristics are emphasized instead of the physician or disease-centered approaches where the doctor makes most decisions. Patient-centeredness focuses on three core values: “First, considering patients’ needs, wants, perspectives and individual experiences. Next, offering patients opportunities to provide input into and participate in their care; and finally, enhancing partnership and understanding in the patient–physician relationship” [2, 3]. Patient-centered care is determined by the quality of the communication between doctors and patients. At its core, patient-centeredness envelops therapeutic relationships based on enhanced communication and trust.

There are numerous studies demonstrating that patient-centered care improves patient satisfaction, quality of care, and health outcomes while decreasing discrepancies in health care and health care costs [4–13].

The burden of non-communicable diseases (NCDs) is widespread and international efforts are focusing on improving health outcomes attributed to them [14]. NCDs cause on average 80% of total deaths in Trinidad and Tobago according to a WHO NCD Country Profile in 2018 [15]. Numerous studies have documented that Trinidad and Tobago (T&T) is not doing well with managing NCDs [16–20]. Could patient-centred consultations offer, in part, the solution? The positive impact of patient-centeredness in consultations for chronic disease care has been described [21]. For example, a T&T study which used a patient-centered approach with poorly controlled diabetic patients demonstrated statistically significant reductions in HbA1c [13].

There is a paucity of studies on the consultation in the English-speaking Caribbean (ESC). In 1998 a paper reported on the benefit of a Continuing Medical Education (CME) intervention for Trinidadian physicians on patient satisfaction of primary care consultations. Since then, there has been no further published work in the ESC studying the consultation in detail [22].

The evidence is less clear on the value of longer consultation times. The Patient Perception of Patient-Centeredness (PPPC) questionnaire was created to measure the extent to which the patients believed the clinician was patient-centered [23]. Another construct, patient enablement index (PEI), has been used as a measure of quality in the primary care setting with good reliability and validity [1, 24]. Given the burden of NCDs and the importance of the consultation in contributing to success in combatting NCDs, the objective of this study was to determine whether patients receiving care for NCDs perceived their consultation to be patient centered or felt enabled as measured by the PPPC and PEI. The consultation length was also determined.

## Methods

### Study design & location

This was a cross-sectional study at 4 primary care health centres (referred to as #1 - #4 in the analysis and text). They are located, not in the numerical order outlined, at St. Joseph, Tacarigua, Macoya and Arouca. They are all part of the St. Joseph Cluster of health centres in the North Central Regional Health Authority (NCRHA) in Trinidad. The survey was conducted between 3rd October and 22nd November 2018.

A meeting with the nurses and staff was conducted prior to the start of data collection at each health centre. They were informed on the nature of the study and given the pertinent information needed for the completion of this study. A separate meeting was held with clinic physicians to explain the nature of the study.

On clinic days, patients were allocated a number which represents the order in which they will be seen. Consecutive patients were invited to participate in the study. No two patients were included from the same household.

To be included patients had to be at least 18 years, English-speaking and be attending the health centre for an NCD. Patients were excluded from participating in the study if there was any intellectual disability or cognitive impairment, or if they were experiencing an unstable psychiatric disorder. 

Any patients with cognitive impairment and intellectual disability are assessed by the doctor and referred to an appropriate clinic, if they were not already enrolled. Such patients are usually accompanied by a relative or caretaker.  

### Data collection instrument

The data collection instrument contained 4 domains: first, demographics, including age, gender, marital status, religion, ethnicity, education level, household income and employment status; secondly, information pertinent to the consultation including the number of medical problems to discuss, the number of interruptions to the consultation, medical condition(s), the number of medications, doctor familiarity and self-perceived general health status. These questions were derived as part of factors associated with the quality of the consultation from previous studies [1, 3, 9, 22, 25].

### The patient perception of patient-centeredness (PPPC) survey

The third part of the questionnaire was the PPPC survey. The PPPC average has been proven to correlate with a score covering the above three core values of patient-centeredness, namely ‘exploring the disease and illness experience’, ‘understanding the whole person’, and ‘finding common ground’ [23]. The questionnaire contains 4 questions pertaining to component 1 (items 1–4), 1 question for component 2 (item 14) and 9 questions for component 3 (items 5–13). Each of the 14 questions has a 4-point Likert scale for responses. The sum of the responses divided by 14 is the final score. The final score ranges from 1 to 4; the higher the score, the more patient-centered the consultation. This tool was identified in a systematic review as one of the 2 best instruments to measure the patient’s perception of patient-centeredness in the consultation [26]. The other instrument was the Consultation Care Measure which had 21 items. The PPPC survey was chosen since it was equal in assessing the patient perception of patient-centeredness but more efficient with 14 items.

### The PEI

The PEI score is an outcome measure of patient-centeredness. This means that the more patient-centered a consultation is, “the greater the enablement of the patient to cope with life, understand and cope with their illness, and keep themselves healthy as a result of their healthcare”. Enablement was shown to be associated significantly with “interest in effect on life, health promotion, and a positive approach” [1]. The PEI questionnaire consists of six questions. Responses are given a score based on a Likert scale ranging from “much better” (2), “better” (1), “same or less” (0) and “not applicable” (0). The maximum score is 12 [24].

### Measurement of consultation length

The consultation length has been studied as a measure of patient-centeredness [27, 28]. The consultation length was measured by one of the interviewers as the patients entered and exited the doctor’s room with a stopwatch.

### Pilot testing of instrument

Pilot testing was performed at the Arouca Health Centre with 10 patients attending an NCD clinic. After the pilot, the following adjustments were made: No change in the wording of the instrument was required, however the measurement of the length of consultations was carried out by the interviewers instead of the doctor. A copy of the final instrument is available in Additional File 1.

### Data collection & analysis

The consultation length was measured by an interviewer using a stopwatch outside the room prior to approaching the patient. The interviewers were volunteer medical doctors who were trained to conduct the consenting process and to administer the instrument. Each patient was approached after they exited the consultation room, the study, and the consent information were explained. Once consent was obtained the questionnaire was administered in a face-to-face interview.

The data collected was analysed using Statistical Package for the Social Sciences (SPSS) Version 23. The main outcomes determined were the mean and SD, median, and mode of the consultation length, PPPC score and PEI score. The frequencies of possible associated factors were tabulated against the ranges for these scores. The associations between patient factors and PEI scores, PPPC Average and consultation length were determined by using the Mann-Whitney U-test and Kruskal-Wallis test. The correlation between continuous variables was tested with the bivariate Spearman’s correlation coefficient (ρ). A *p*-value of less than 0.05 was deemed statistically significant. Post-hoc pair-wise comparisons were used to explore significant associations revealed by Kruskal-Wallis tests. All post-hoc tests conducted were Bonferroni corrected to reduce type 1 error inflation.

### Ethical issues

Approvals were attained from the Faculty of Medical Sciences’ Ethics Committee (approval # CEC 692/08/18), and the North Central Regional Health Authority, Trinidad and Tobago. All participants provided written informed consent to participate. Seven participants had not completed any formal education (in Table 1); however, these participants were all functionally literate and were able to understand the consent forms, which were read to them by the research assistant. They were all able to sign the consent form.

**Table 1:** Socio-demographics of the participants and characteristics of their doctor-patient encounter in a population of patients with non-communicable diseases in Trinidad

Variable	N = 180	N (% )
Sex	Male	44 (24.4)
	Female	136 (75.6)
Age	18–35	3 (1.7)
	36–45	12 (6.7)
	46–55	20 (11.1)
	56–65	54 (30)
	> 65	91 (50.6)
Ethnicity	East Indian	94 (52.2)
	African	50 (27.8)
	Mixed (East Indian and African)	20 (11.1)
	Mixed (Other)	14 (7.8)
	Caucasian	2 (1.1)
Religion	Christian	109 (60.6)
	Hindu	48 (26.7)
	Muslim	8 (4.4)
	Baptist	4 (2.2)
	Jehovah’s Witness	2 (1.1)
	Seventh Day Adventist	4 (2.2)
	Other	5 (2.8)
Marital Status	Married or Co-habitant	92 (51.1)
	Widowed or divorced or separated	61 (33.9)
	Single	27 (15)
Education level	None	7 (3.9)
	Primary School	89 (49.4)
	Secondary School	67 (37.2)
	University or tertiary level	17 (9.4)
Household monthly Income	<$6000	118 (65.6)
	$6000–10,000	43 (23.9)
	$10,001–15,000	12 (6.7)
	15,001–20,000	4 (2.2)
	> $20,000	3 (1.7)
Employment status	Employed	44 (24.4)
	Unemployed	136 (75.6)
Medical Problems to discuss	0	9 (5)
	1	56 (31.1)
	2	62 (34.4)
	More than 2	53(29.4)
Number of Consultation interruptions	0	144 (80)
	1	22 (12.2)
	2	9 (5)
	More than 2	5 (2.8)
Number of Medications	1	15 (8.3)
	2	31 (17.2)
	3	35 (19.4)
	4	42 (23.3)
	More than 4	57 (31.7)
General Health Rating	Poor	8 (4.4)
	Fair	57 (31.7)
	Good	86 (47.8)
	Very Good	22 (12.2)
	Excellent	7 (3.9)
Doctor familiarity	Not at all	62 (34.4)
	Somewhat	52 (28.9)
	Well	36 (20)
	Very well	30 (16.7)

## Results

A total of 180 patients were enrolled. The response rate was 82.5%, ranging from 70 to 100% at the 4 centres.

### Demographics

Participants were 75.6% female; the majority were aged over 65 (50.6%). 52.2% were Indo-Trinidadian, and 60.6% Christian. 51.1% were married or had a co-habitant, 33.9% were widowed, separated, or divorced and 15% were single. 49.4% achieved primary school level, and 37.2% secondary school level. The household monthly income was distributed as 65.6% earning less than $6000, and 23.9% between $6000–10,000 (TTD), (1 USD = 6.75 TTD). 75.6% were unemployed. The sample’s socio-demographic characteristics are shown in Table 1.

### Consultation parameters

34.4% had 2 medical problems to be discussed, 31.7% used more than 4 medications, and 47.8% gave a general health rating as ‘good’. 34.4% did not know the doctor at all. Most consultations had no interruptions (80%). See Table 1.

### Consultation length

**Fig. 1:**
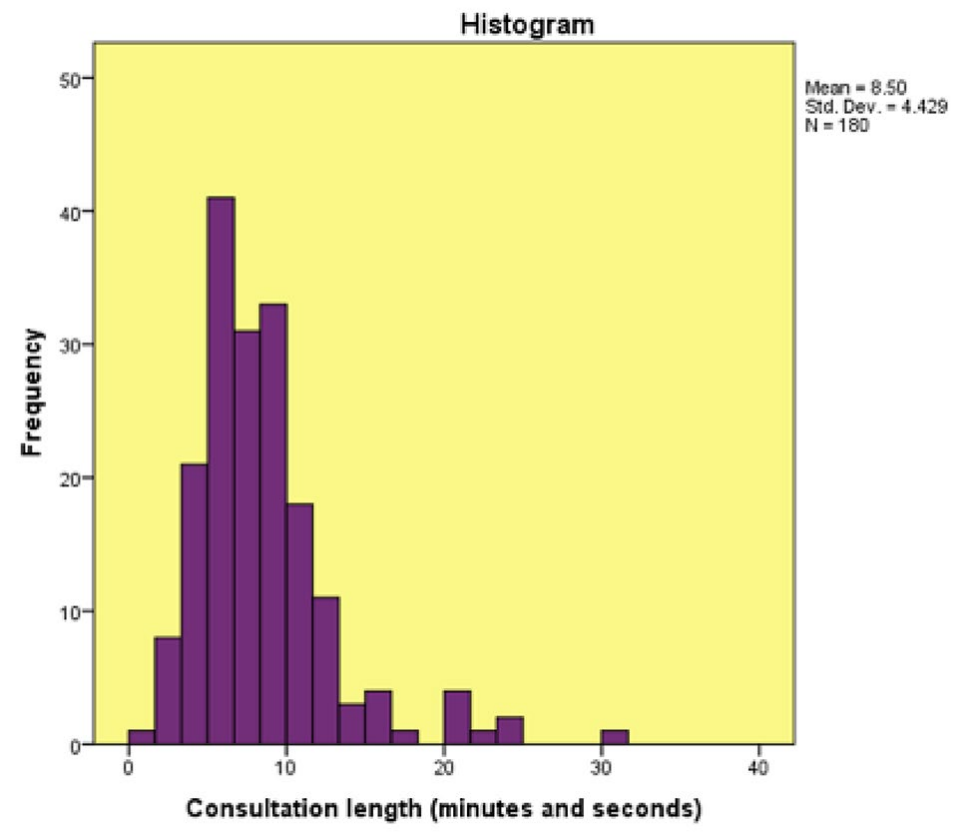
Histogram of Consultation length and frequency in a population of patients with non-communicable diseases in Trinidad

The average, median and modal consultation lengths were 8.5, 7.74 and 10 min respectively, with a range of 1.32–31.22 min. The interquartile range was 4.28. The distribution may be seen in Fig. 1.

### PPPC average

**Fig. 2:**
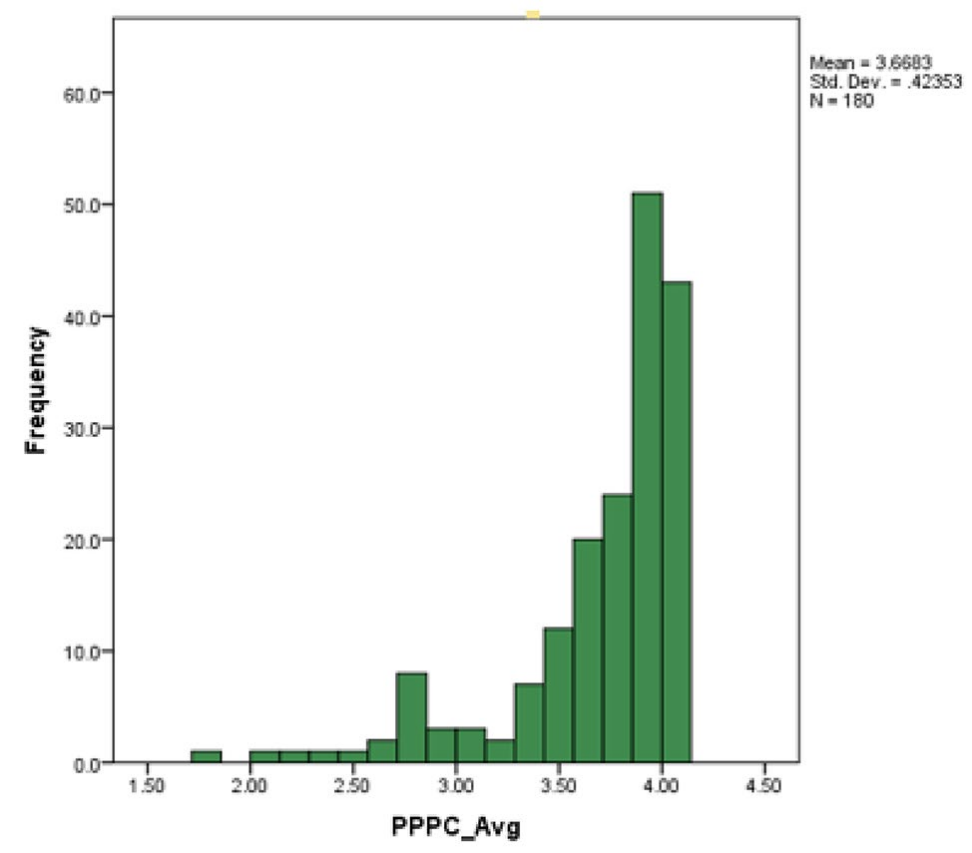
Histogram of PPPC Average and frequency in a population of patients with non-communicable diseases in Trinidad

The PPPC was distributed between 1.79 and 4.00. The inter quartile range was 0.36 and the median was 3.86. The mean was 3.67 with a standard deviation of 0.42. The PPPC was negatively skewed (See Fig. 2).

### PEI score

**Fig. 3:**
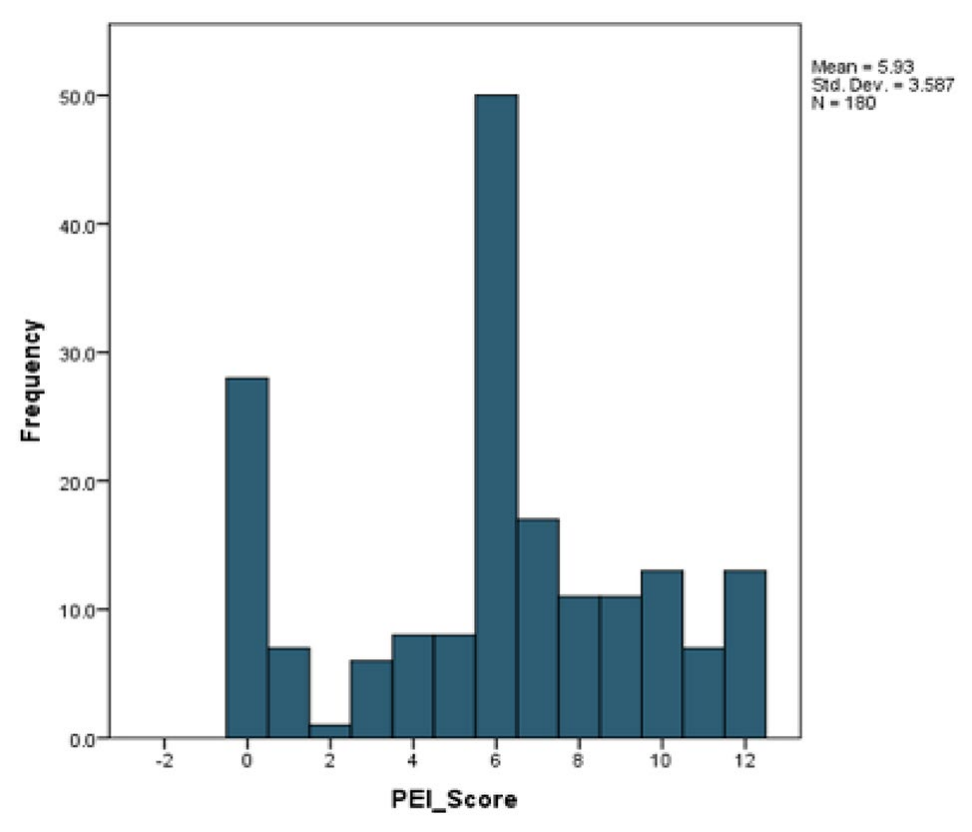
Histogram of PEI Score and frequency in a population of patients with non-communicable diseases in Trinidad

The PEI score ranged between 0 and 12. The interquartile range was 4 and the median was 6.00. The mean was 5.93 and the standard deviation was 3.59. The distribution may be seen in Fig. 3.

### Correlations between PPPC score, patient demographics, and consultation parameters

This analysis revealed no statistical differences in the patient perception of patient-centeredness when PPPC scores were assessed by gender, employment status, age, ethnicity, religion, marital status, education, income, number of medical problems to discuss, non-communicable disease, number of medications used and general health rating. The PPPC was determined to be lower in those with stroke (3.10 ± 0.39, 3.07) (Mean ± SD, Median) than those without stroke (3.68 ± 0.42, 3.86) with a *p*-value of 0.022. The PPPC was significantly higher in health centre 4 (3.76 ± 0.41, 3.93) than health centre 1 (3.57 ± 0.49, 3.79) with *p*-value of 0.017. The PPPC was higher in those with more than 2 consultation interruptions (3.99 ± 0.03, 4.00) than those with one consultation interruption (3.49 ± 0.55, 3.64) with *p* = 0.015. The PPPC was significantly higher (*p* = 0.015) with continuity of care. The PPPC in those patients that knew the doctor very well (3.79 ± 0.35, 3.93) was higher than those who did not know the doctor at all (3.57 ± 0.47, 3.71). See Table 2.

**Table 2:** Comparison of PPPC Average and Patient characteristics in a population of patients with non-communicable diseases in Trinidad

Variables		PPPC Average (mean ± SD, Median)	Significance value
Gender*	Female	3.68 ± 0.43, 3.86	*p* = 0.655
	Male	3.64 ± 0.42, 3.86	
Employment*	Employed	3.71 ± 0.39, 3.86	*p* = 0.311
	Unemployed	3.65 ± 0.43, 3.86	
Stroke*	No	3.68 ± 0.42, 3.86	*p* = 0.022
	Yes	3.10 ± 0.39, 3.07	
**Age**	18–45	3.54 ± 0.68, 3.86	*p* = 0.509
	46–55	3.51 ± 0.58, 3.68	
	56–65	3.68 ± 0.37, 3.79	
	More than 65	3.72 ± 0.35, 3.86	
**Ethnicity**	East Indian	3.69 ± 0.41, 3.86	*p* = 0.329
	African	3.63 ± 0.45, 3.79	
	Mixed (East Indian and African)	3.73 ± 0.43, 3.93	
	Other	3.61 ± 0.40, 3.75	
**Religion**	Christian	3.69 ± 0.41, 3.86	*p* = 0.453
	Hindu	3.63 ± 0.47, 3.82	
	Other	3.66 ± 0.42, 3.79	
**Marital status**	Married/ Co-habitant	3.68 ± 0.44, 3.86	*p* = 0.247
	Single	3.75 ± 0.35, 3.86	
	Widowed/ Divorced/ Separated	3.62 ± 0.43, 3.79	
**Education**	None	3.67 ± 0.44, 3.86	*p* = 0.685
	Primary school	3.70 ± 0.34, 3.79	
	Secondary school	3.64 ± 0.47, 3.86	
	University/ Tertiary level	3.66 ± 0.59, 3.93	
**Income**	Less than $6000	3.63 ± 0.44, 3.79	*p* = 0.070
	$6000–10,000	3.74 ± 0.44, 3.79	
	More than $10,000	3.76 ± 0.38, 3.93	
**Health Centre**	Health Centre 1	3.57 ± 0.49, 3.79	Vs Health Centre 4 *p* = 0.017
	Health Centre 2	3.65 ± 0.38, 3.79	
	Health Centre 3	3.69 ± 0.39, 3.86	
	Health Centre 4	3.76 ± 0.41, 3.93	
**Medical problems to discuss**	0	3.48 ± 0.67, 3.64	*p* = 0.495
	1	3.67 ± 0.42, 3.86	
	2	3.64 ± 0.46, 3.86	
	More than 2	3.74 ± 0.33, 3.86	
**Number of consultation interruptions**	0	3.68 ± 0.40, 3.86	Vs more than 2 *p* = 0.015
	1	3.49 ± 0.55, 3.64	
	2	3.66 ± 0.38, 3.79	
	More than 2	3.99 ± 0.03, 4.00	
**Number of medications used**	1	3.59 ± 0.53, 3.86	*p* = 0.473
	2	3.62 ± 0.44, 3.79	
	3	3.64 ± 0.48, 3.86	
	4	3.70 ± 0.41, 3.89	
	More than 4	3.71 ± 0.36, 3.86	
**General health rating**	Poor	3.78 ± 0.22, 3.82	*p* = 0.393
	Fair	3.55 ± 0.57, 3.86	
	Good	3.74 ± 0.34, 3.86	
	Very good	3.66 ± 0.25, 3.68	
	Excellent	3.70 ± 0.44, 3.86	
**Doctor familiarity**	Not at all	3.57 ± 0.47, 3.71	Vs Very well *p* = 0.015
	Somewhat	3.67 ± 0.44, 3.86	
	Well	3.74 ± 0.34, 3.86	
	Very well	3.79 ± 0.35, 3.93	

Mann-Whitney U-test as indicated*. All others Kruskal-Wallis test

### Correlations between PEI score, patient demographics and consultation parameters

This analysis revealed no statistically significant differences in the PEI by gender, employment status, age, ethnicity, religion, marital status, education, income, number of medical problems to discuss, number of consultation interruptions, number of medications used, general health rating and doctor familiarity. The PEI of those without heart disease (6.24 ± 3.61, 6.00) (Mean ± SD, Median) was significantly (*p* = 0.022) higher than those with heart disease (4.45 ± 3.15, 6.00). The PEI score of those in health centre #1 (6.58 ± 3.76, 6.65) was significantly (*p* = 0.049) higher in health centre #3 (4.96 ± 3.74, 6.00). The PEI score in health centre #4 (6.96 ± 3.57, 6.00) was significantly (*p* = 0.032) higher than health centre #3 (4.96 ± 3.74, 6.00). See Table 3.

**Table 3:** Comparison of PEI Score and Patient characteristics in a population of patients with non-communicable diseases in Trinidad

Variable		PEI Score (mean ± SD, Median)	Significance value
Gender*	Female	6.10 ± 3.52, 6.00	*p* = 0.305
	Male	5.41 ± 3.77, 6.00	
Employment*	Employed	6.48 ± 3.29, 6.00	*p* = 0.311
	Unemployed	5.76 ± 3.67, 6.00	
Heart disease*	No	6.24 ± 3.61, 6.00	*p* = 0.020
	Yes	4.45 ± 3.15, 6.00	
**Age**	18–45	6.67 ± 3.74, 7.00	*p* = 0.603
	46–55	5.40 ± 3.82, 5.50	
	56–65	6.06 ± 3.80, 6.00	
	More than 65	5.86 ± 3.41, 6.00	
**Ethnicity**	East Indian	5.90 ± 3.47, 6.00	*p* = 0.510
	African	5.56 ± 3.64, 6.00	
	Mixed (East Indian and African)	6.85 ± 3.87, 6.50	
	Other	6.13 ± 3.88, 7.00	
**Religion**	Christian	6.24 ± 3.61, 6.00	*p* = 0.263
	Hindu	5.08 ± 3.51, 6.00	
	Other	6.26 ± 3.51, 6.00	
**Marital status**	Married/ Co-habitant	5.70 ± 3.84, 6.00	*p* = 0.634
	Single	5.52 ± 2.99, 6.00	
	Widowed/ Divorced/ Separated	5.95 ± 3.43, 6.00	
**Education**	None	4.29 ± 3.86, 6.00	*p* = 0.061
	Primary school	6.46 ± 3.37, 6.00	
	Secondary school	5.84 ± 3.85, 6.00	
	University/ Tertiary level	4.24 ± 3.01, 5.00	
**Income**	Less than $6000	5.92 ± 3.76, 6.00	*p* = 0.946
	$6000–10,000	5.79 ± 3.37, 6.00	
	More than $10,000	6.32 ± 3.09, 6.00	
**Health Centre**	Health Centre 1	6.58 ± 3.76, 6.65	
	Health Centre 2	5.24 ± 2.92, 6.00	
	Health Centre 3	4.96 ± 3.74, 6.00	Vs Health Centre 1 *p* = 0.049
	Health Centre 4	6.96 ± 3.57, 6.00	Vs Health Centre 3 *p* = 0.032
**Medical problems to discuss**	0	5.22 ± 3.19, 6.00	*p* = 0.178
	1	6.66 ± 3.74, 7.00	
	2	5.50 ± 3.64, 6.00	
	More than 2	5.79 ± 3.64, 6.00	
**Number of consultation interruptions**	0	5.93 ± 3.48, 6.00	*p* = 0.920
	1	5.77 ± 4.42, 6.00	
	2	5.67 ± 4.09, 6.00	
	More than 2	7.20 ± 2.17, 6.00	
**Number of medications used**	1	6.73 ± 4.01, 6.00	*p* = 0.050
	2	6.16 ± 3.45, 6.00	
	3	7.09 ± 3.58, 7.00	
	4	6.07 ± 3.74, 6.00	
	More than 4	4.79 ± 3.21, 6.00	
**General health rating**	Poor	7.38 ± 2.26, 6.50	*p* = 0.472
	Fair	5.11 ± 3.46, 6.00	
	Good	6.14 ± 3.83, 6.14	
	Very good	6.50 ± 3.07, 6.00	
	Excellent	6.71 ± 3.68, 6.00	
**Doctor familiarity**	Not at all	5.71 ± 3.83, 6.00	*p* = 0.716
	Somewhat	6.17 ± 3.81, 6.50	
	Well	5.58 ± 3.65, 6.00	
	Very well	6.40 ± 2.53, 6.00	

Mann-Whitney U-test as indicated*. All others Kruskal-Wallis test

### Correlations between consultation duration, patient demographics and consultation parameters

This analysis revealed no statistical difference in the consultation length when compared by gender, employment, medical conditions, age, ethnicity, religion, marital status, income, number of medical problems to discuss, number of medications used, general health rating and doctor familiarity. There was a statistically significant difference in consultation length between tertiary level education (11.32 ± 5.78, 10.10) (Mean ± SD, Median) and primary school level (7.59 ± 3.11, 6.83) with *p* = 0.044. Health centre #4 (11.39 ± 6.30, 9.63) had significantly longer consultation than health centre #1 (7.85 ± 3.21, 7.55) and health centre #2 (6.14 ± 2.42, 5.93) where *p* = 0.017 and *p* < 0.001, respectively. The consultation length in health centre #3 (8.61 ± 3.06, 8.37) was significantly longer than health centre #2 (6.14 ± 2.42, 5.93) with *p* = 0.001. The consultation length in those with no consultation interruptions (7.96 ± 3.72, 7.41) was found to be significantly shorter than those with two consultation interruptions (12.58 ± 7.37, 11.17) with *p* = 0.032.

These findings are illustrated in Table 4.

**Table 4:** Comparison of Consultation length and Patient Characteristics in a population of patients with non-communicable diseases in Trinidad

Variable			Consultation length (mean ± SD, Median)	Significance value
Gender*	Female		8.36 ± 4.30, 7.53	*p* = 0.478
	Male		8.91 ± 4.83, 8.40	
Employment*	Employed		8.43 ± 3.06, 8.18	*p* = 0.366
	Unemployed		8.52 ± 4.80, 7.55	
**Age**	18–45		10.17 ± 2.83, 9.87	*p* = 0.509
	46–55		8.92 ± 4.36, 7.54	
	56–65		7.60 ± 4.50, 6.18	
	More than 65		8.66 ± 4.56, 8.13	
**Ethnicity**	East Indian		8.35 ± 4.67, 6.78	*p* = 0.510
	African		8.57 ± 3.74, 8.21	
	Mixed (East Indian and African)		8.09 ± 5.15, 9.37	
	Other		6.66 ± 3.25, 6.54	
**Religion**	Christian		9.10 ± 5.11, 8.27	*p* = 0.272
	Hindu		7.56 ± 2.93, 6.71	
	Other		7.61 ± 2.96, 8.00	
**Marital status**	Married/ Co-habitant		8.34 ± 4.13, 7.89	*p* = 0.187
	Single		9.49 ± 4.80, 8.78	
	Widowed/ Divorced/ Separated		8.30 ± 4.71, 6.70	
**Education**	None		11.64 ± 6.51, 10.00	Vs *P*rimary school *p* = 0.044
	Primary school		7.59 ± 3.11, 6.83	
	Secondary school		8.66 ± 4.91, 8.00	
	University/ Tertiary level		11.32 ± 5.78, 10.10	
**Income**	Less than $6000		8.37 ± 3.93, 7.68	*p* = 0.968
	$6000–10,000		8.94 ± 5.87, 8.00	
	More than $10,000		8.27 ± 3.73, 7.60	
**Health Centre**	Health Centre 1		7.85 ± 3.21, 7.55	Vs Health centre 4 *p* = 0.017
	Health Centre 2		6.14 ± 2.42, 5.93	Vs Health Centre 4 *p* < 0.001
	Health Centre 3		8.61 ± 3.06, 8.37	Vs Health Centre 2 *p* = 0.001
	Health Centre 4		11.39 ± 6.30, 9.63	
**Medical problems to discuss**	0		9.13 ± 7.67, 6.72	*p* = 0.568
	1		8.16 ± 3.50, 8.07	
	2		8.25 ± 4.19, 7.10	
	More than 2		9.03 ± 4.93, 8.60	
**Number of consultation interruptions**	0		7.96 ± 3.72, 7.41	Vs 2 *p* = 0.032
	1		9.97 ± 5.75, 9.17	
	2		12.58 ± 7.37, 11.17	
	More than 2		10.11 ± 6.27, 8.40	
**Number of medications used**	1		8.38 ± 4.50, 7.00	*p* = 0.712
	2		8.83 ± 3.69, 8.37	
	3		8.25 ± 3.81, 7.75	
	4		7.86 ± 3.82, 6.52	
	More than 4		8.97 ± 5.50, 8.33	
**General health rating**	Poor		8.61 ± 1.87, 9.00	*p* = 0.072
	Fair		9.18 ± 4.25, 8.00	
	Good		8.27 ± 4.66, 7.04	
	Very good		8.55 ± 4.84, 7.25	
	Excellent		5.50 ± 2.74, 5.20	
**Doctor familiarity**	Not at all		8.16 ± 3.71, 8.02	*p* = 0.380
	Somewhat		7.98 ± 3.97, 7.40	
	Well		8.84 ± 5.69, 6.49	
	Very well		9.69 ± 4.79, 8.78	

Mann-Whitney U-test as indicated*. All others Kruskal-Wallis test

### Predictors of PPPC and PEI scores

Based on other reports from the literature the consultation length was grouped into 0–9 min, 10–15 min, and more than 15 min [29]. Analysis suggests that these groupings of consultation times did not have any statistical association with the PEI or PPPC Scores. There were no statistical associations found between the PPPC or PEI and any of the consultation parameters (Table 5).

**Table 5:** Consultation Parameters and Patient-centered Measures in a population of patients with non-communicable diseases in Trinidad*

Characteristic	No. of patients (%)	PEI Score (SD)	PPPC Average (SD)	Consultation Length/minutes (SD)
**Consultation length (minutes)**				
0–9	130 (72.2)	5.88 (3.56)	3.65 (0.422)	
10–15	37 (20.6)	6.59 (3.66)	3.70 (0.074)	
> 15	13 (7.2)	4.62 (3.48)	3.69 (0.377)	
**Doctor familiarity**				
Not at all	62 (34.4)	5.71 (3.83)	3.57 (0.465)	8.16 (3.71)
Somewhat	52 (28.9)	6.17 (3.81)	3.67 (0.445)	7.98 (3.97)
Well	36 (20.0)	5.58 (3.65)	3.74 (0.341)	8.84 (5.69)
Very well	30 (16.7)	6.40 (2.53)	3.79 (0.350)	9.69 (4.79)
**Consultation Interruptions**				
0	144 (80.0)	5.93 (3.48)	3.68 (0.405)	7.96 (3.72)
1	22 (1.2)	5.77 (4.42)	3.49 (0.547)	9.97 (5.75)
More than 1	14 (7.8)	6.21 (3.51)	3.78 (0.341)	11.7 (6.85)
**General health rating**				
Poor	8 (4.4)	7.38 (2.26)	3.78 (0.224)	8.61 (1.87)
Fair	57 (31.7)	5.11 (3.46)	3.55 (0.565)	9.18 (4.25)
Good	86 (47.8)	6.14 (3.83)	3.74 (0.343)	8.27 (4.66)
Very good	22 (12.2)	6.5 (3.07)	3.66 (0.254)	8.55 (4.84)
Excellent	7 (3.9)	6.71 (3.68)	3.7 (0.439)	5.5 (2.74)
**Number of medications**				
1	15 (8.3)	6.73 (4.01)	3.59 (0.53)	8.38 (4.5)
2	31 (17.2)	6.16 (3.45)	3.62 (0.438)	8.83 (3.69)
3	35 (19.4)	7.09 (3.58)	3.64 (0.479)	8.25 (3.81)
4	42 (23.3)	6.07 (3.74)	3.7 (0.41)	7.86 (3.82)
More than 4	57 (31.7)	4.79 (3.21)	3.71 (0.364)	8.97 (5.5)
**Medical problems to discuss**				
0	9 (5.0)	5.22 (3.19)	3.48 (0.668)	9.13 (7.67)
1	56 (31.1)	6.66 (3.74)	3.67 (0.418)	8.16 (3.5)
2	62 (34.4)	5.50 (3.64)	3.64 (0.455)	8.25 (4.19)
More than 2	53 (29.4)	5.79 (3.39)	3.74 (0.329)	9.03 (4.93)

*All analyses: *p-*values > 0.05

### Spearman’s correlations

The correlation coefficient ρ = 0.408 was significant, *p* < 0.001 showing that PPPC Average correlated positively with PEI Score. The consultation length significantly correlated with the PPPC Average with the correlation coefficient ρ *=* 0.168, *p* = 0.025. The PEI Score did not correlate with consultation length significantly with the correlation coefficient ρ = 0.092 (*p* = 0.220).

## Discussion

This study was able to determine the patient perception of patient-centeredness (PPPC), the patient enablement (PEI) and the length of the consultation, among a sample of primary care patients seeking NCD care in North-central Trinidad. From our literature review this was the first time such a study was attempted in the English-speaking Caribbean.

### **PPPC**- **how does T&T compare internationally?**

Our PPPC Average score 3.67 (± 0.42) compared favourably with a reported score from Japan, 3.19 (± 0.48) [30] and better than a score from Canada, 1.5 (± 0.37) [4].

There was some local variation in PPPC scores, for example, the PPPC Average was significantly higher in health centre #4 compared with health centre #1. We postulate that this is because health centre 4 has a lower patient load and therefore could achieve longer consultations (11.39 min vs. 7.85 min respectively). The association between consultation time and PPPC has been shown in previous studies, such as by Howie et al. [1]. This reinforces the point that adequate time and continuity of care are both necessary for patient-centeredness to be achieved.

The PPPC Average was lower in patients with a stroke. This is possibly because patients with stroke either need or desire more time and attention. They may also have been experiencing a mood, articulation or cognitive disorder which may lead to lower PPPC scores.

Continuity of care results in greater patient-centeredness [31]. In our study, this was seen with a significantly higher PPPC average in those that knew the doctor ‘very well’ versus those who ‘did not know the doctor at all’. A higher PPC score was seen in those with 2 or more interruptions. Consultation times were however longer in those with reported interruptions suggesting that the interruptions did not detract from the overall patient-centered experience.

### PEI score- how does T&T compare internationally?

The PEI in our study was determined to be 5.93. In a brief review this was third out of the 9 reports from several countries, including Portugal with the highest PEI score of 7.85 (SD = 3.13) [32], and Sweden with the lowest PEI score of 3.48 (SD = 3.21) [33] and similar to ours, a score from French Canada where the mean PEI score was 5.06 (95% CI: 4.30–5.81) [34].

The differences in PEI in the above countries could be explained by varied primary care needs in diverse populations, cultural variances in inclination to report negative responses, dissimilar understandings of the “enablement” concept, confidence in the doctors in primary care, confidence in the health system and its benefits, continuity of care or genuine variations in the quality of delivered care.

The PEI score was lower in those with heart disease, which may be due to those with heart disease requiring more detailed attention with multiple medical conditions and complex medicine regimes. The variations in PEI among the health centres were not correlated with consultation length or PPPC score. Our study did not find a correlation of PEI with consultation time, which was also found to be a non-significant correlating factor in some previous studies [35, 36].

### Consultation length- how does T&T compare internationally?

The consultation length average of 8.5 min determined in this study was almost twice the average consultation length (4.6 min for physicians with special training) of a study conducted in Trinidad 20 years prior [22]. Although not recorded in this study, our experience is that in this cluster of health centres about 2/3 of the doctors have completed post-graduate training in family medicine. Communication and consultation skills are integral parts of this training. There are also monthly teachings in the cluster with updates in management.

The consultation lengths in primary care consultations of 67 countries were reviewed in 2017 [29]. The range of consultation length was between 48 s in Bangladesh and 22.5 min in Sweden. Only 16 countries had consultation lengths on average more than 15 min. The ranking of T&T is 33^rd^ among the 67 countries in this international study.

Short consultation lengths are likely to negatively affect patient care and the workload and pressure of the consulting physician. The variances among countries are explained by factors relating to politics and policy, labour force, access, continuity, comprehensiveness, and management. The least effective consultation length is on average 5 min, which at most consists of a greeting and issuing a prescription. These shorter consultation lengths were shown to result in increasing polypharmacy, misuse of antibiotics and inferior communication with patients [37–39].

In this study, the consultation length in those patients with University or Tertiary level education was higher than those with Primary school level. This may be due to the greater understanding of their condition by the patient, which in turn may likely result in more questioning of, and discussion with, the doctor. Insufficient time in the consultation is an egregious constraint on the delivery of primary care [40]. This is evident by a significant association between shorter consultation and physician burnout. This is postulated to be due to a sense of inadequate personal accomplishment by the doctor in these shorter consultations, which leads to doctors feeling less effective and less capable of managing complex multi-morbid patients [40]. This problem needs to be resolved for these patients who require adequate time for effective management in primary care.

### Strengths and weaknesses of the study

This study was novel to the Caribbean region and can be used as a baseline and to inform future research in the fundamental aspects of patient centeredness in primary care.

We were able to achieve a good response rate, and an adequate sample size (See Additional file 1), using valid and internationally recognized tools. Recall bias associated with cross-sectional studies was not significant in this case since the consultation they just completed was the one evaluated.

The non-English speaking patients were excluded due to the language barrier however during the study only English-speaking patients attended the clinic. There are very few non-English speaking patients in the sample population.

A weakness of this study is that physicians knew they were being observed. This could have influenced how physicians behaved. However, we see that 70% of consultations lasted less than 9 min. If the Hawthorne effect exerted a strong bias, we could expect many more consultations being of longer duration. Weaknesses of the study would include a lack of generalizability of the results to other health centres in Trinidad, the non-response for certain questions in the instrument, especially around the PEI questionnaire where many of the responses were ‘same’, ‘more or less’ or ‘not applicable’. This survey did not measure other factors including physician-related variables that can influence consultation quality, such as fatigue or education.

### Recommendations

The training of health care providers in patient-centeredness has been shown in multiple systematic reviews to improve health outcomes [10, 41–43]. Policy makers need to become aware of these findings. Future postgraduate (PG) education and CME for primary care physicians working in these health centre clusters should focus on the consultation and on quality issues around consultations. Staff should be supported in conducting audits of their practice. There should be further follow up assessment in other health centre clusters of this work with standardization of the PPPC and PEI in this population.

## Conclusion

In general, the results suggest that the encounters at primary health care centres in Trinidad are patient-centered, with attention being paid to the patient’s illness experience, understanding the whole person, and finding common ground. The patients also in general, had a sense of enablement, i.e., being able to cope with life, understand and cope with their illness, and keep themselves healthy because of their healthcare. The consultation length has roughly doubled compared to a study 20 years ago.

### Electronic supplementary material

Below is the link to the electronic supplementary material.

**Additional file 1**

## Data Availability

The datasets used during the current study are not available to the public as additional analysis is ongoing. It is however available from the corresponding author on reasonable request.

## Abbreviations

CME: Continuing Medical Education
ESC: English-speaking Caribbean
NCD: Non communicable disease
NCRHA: North Central Regional Health Authority
PCC: Patient Centered Care
PEI: Patient Enablement Index
PG: Postgraduate
PPPC: Patient Perception of Person Centered Care
SD: Standard Deviation
SPSS: Statistical Package for the Social Sciences
T&T: Trinidad and Tobago
WHO: World Health Organization
