# Supplementary material for: Are primary care consultations in Trinidad patient-centered? A cross-sectional study of patients with non-communicable diseases
Source: BMC Prim Care. 2023 Oct 21;24:215. doi: 10.1186/s12875-023-02149-8 (PMC10589933; doi:10.1186/s12875-023-02149-8)
Supplement: Supplementary file 1 — Additional file 1 [file 12875_2023_2149_MOESM1_ESM.docx]

**ADDITIONAL FILE 1.**

**Sample size calculation**

Cochran formula was used-


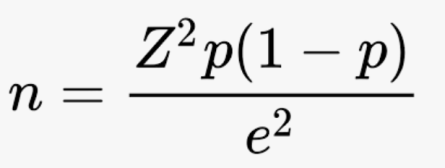


z= 1.96

p = 0.1 (10% of the population had consultations longer than 15 minutes)

q = 1-p = 0.9

e= 0.05

If you substitute values in the above formula, the sample size is **138**

The expected non-response rate is 0.20. Therefore the corrected sample size needed to complete the survey was determined as below.

Formula for Correction of Sample size due to dropout/; non-response rate;

= n x[1/ (1-dropout rate)]

=138 x [1/(1-0.20)]

=172.5

Therefore Sample size = 173 participants.

**Questionnaire**

Please choose one answer to each of the following questions.

Please indicate by ticking (✓) the appropriate box.

All information shared will be treated with the strictest of confidence.

## SECTION 1. DEMOGRAPHICS

| 1. How old are you?   1. 18 to 35 2. 36 to 45 3. 46 to 55 4. 56 to 65 5. More than 65 | 2. What is your sex?   1. Female 2. Male |
| --- | --- |
| 3. What is your ethnicity?   1. East Indian 2. African 3. Mixed (East Indian and African) 4. Mixed (Other than East Indian and African) 5. Caucasian 6. Chinese 7. Syrian/Lebanese 8. Indigenous 9. Other | 4. What is your religion?   1. Christian 2. Hindu 3. Muslim 4. Baptist 5. Jehovah’s Witness 6. Seventh Day Adventist 7. Other |
| 5. What is your marital status?   1. Married/ co-habitant 2. Single 3. Widowed/divorced/separated 4. In a relationship | 6. What is your completed level of education?   1. None 2. Primary School 3. Secondary School 4. University or Tertiary level 5. Post-Graduate |

| 7. What is your household’s combined monthly income?   1. Less than $6,000 2. $6,000 to 10,000 3. $10,001 to 15,000 4. $15,001 to 20,000 5. More than $20,000 | 8. Are you presently employed (partial/fulltime)?   1. Yes 2. No |
| --- | --- |

## SECTION 2. CONSULTATION/ HEALTH CONDITION

| 9. How many medical problems did you want to discuss?   1. 0 2. 1 3. 2 4. More than 2 | 10. How many times was your consultation interrupted?   1. 0 2. 1 3. 2 4. More than 2 |
| --- | --- |
| 11. What medical condition(s) do you suffer from?   1. Diabetes 2. Pre-diabetes 3. Chronic respiratory disease (Asthma/ COPD) 4. Cancer 5. Heart disease 6. Stroke 7. Hypertension 8. Hypercholesterolaemia 9. Overweight/Obesity | Please tick as many as are appropriate.   1. Thyroid disorder 2. Arthritis 3. Blood dyscrasia (e.g. SCD, Thalassemia etc.) 4. Seizure Disorder 5. Dementia 6. Prostate disease 7. Psychiatric Illness (e.g. Depression, Anxiety, Bipolar) 8. Other |
| 12. How many medications do you use presently?   1. 1 2. 2 3. 3 4. 4 5. More than 4 | 13. How will you rate your health in general?   1. Poor 2. Fair 3. Good 4. Very Good 5. Excellent |
| 14. To what extent do you know the doctor?   1. Not at all 2. Somewhat 3. Well 4. Very well |  |
| **SECTION 3. PATIENT PERCEPTION OF PATIENT CENTEREDNESS** | |
| 1. To what extent was your main problem(s) discussed?  4)Completely  3)Mostly  2)A little  1)Not at all | 2. Would you say that your doctor knows that this was one of your reasons for coming today?  4)Yes  3)Probably  2)Unsure  1)No |
| 3. To what extent did the doctor understand the importance of your reason for coming in today?  4)Completely  3)Mostly  2)A little  1)Not at all | 4. How well do you think your doctor understood you today?  4)Very well  3)Well  2)Somewhat  1)Not at all |
| 5. How satisfied were you with the discussion of your problem?  4)Very satisfied  3)Satisfied  2)Somewhat satisfied  1)Not satisfied | 6. To what extent did the doctor explain this problem to you?  4)Completely  3)Mostly  2)A little  1)Not at all |
| 7. To what extent did you agree with the doctor’s opinion about the problem?  4)Completely  3)Mostly  2)A little  1)Not at all | 8. How much opportunity did you have to ask your questions?  4)Very much  3)A fair amount  2)A little  1)Not at all |
| 9. To what extent did the doctor ask about your goals for treatment?  4)Completely  3)Mostly  2)A little  1)Not at all | 10. To what extent did the doctor explain treatment?  4)Very well  3)Well  2)Somewhat  1)Not at all |
| 11. To what extent did the doctor explore how manageable this (treatment) would be for you? He/she explored this.  4)Completely  3)Mostly  2)A little  1)Not at all | 12. To what extent did you and the doctor discuss your respective roles? (Who is responsible for making decisions and who is responsible for what aspects of your care?)  4)Completely  3)Mostly  2)A little  1)Not at all |
| 13. To what extent did the doctor encourage you to take the role you wanted in your own care?  4)Completely  3)Mostly  2)A little  1)Not at all | 14. How much would you say that this doctor cares about you as a person?  4)Very much  3)A fair amount  2)A little  1)Not at all |

|  |
| --- |
|  |
|  |

## SECTION 4. PATIENT ENABLEMENT INSTURMENT

| As a result of your visit to the doctor today do u feel you are | Scale | | | |
| --- | --- | --- | --- | --- |
|  | Much better | Better | Same orLess | Not Applicable |
| 1. Able to cope with life? | 2 | 1 | 0 | 0 |
| 1. Able to understand your illness? | 2 | 1 | 0 | 0 |
| 1. Able to cope with your illness? | 2 | 1 | 0 | 0 |
| 1. Able to keep yourself healthy? | 2 | 1 | 0 | 0 |
|  | **Much more** | **More** | **Same or Less** | **Not Applicable** |
| 1. Confident about your health? | 2 | 1 | 0 | 0 |
| 1. Able to help yourself? | 2 | 1 | 0 | 0 |

| **LENGTH OF CONSULTATION**  **_____________Minutes ____________Seconds** |  |
| --- | --- |

***Your answers will be kept entirely confidential and will not be shown to anyone else.***

***Thank you for your participation!***
